# Genetic assessment of apolipoprotein E polymorphism and PRNP genotypes in rapidly progressive dementias in Pakistan

**DOI:** 10.1080/19336896.2024.2439598

**Published:** 2024-12-09

**Authors:** Urwah Rasheed, Minahil Khalid, Aneeqa Noor, Umar Saeed, Rizwan Uppal, Saima Zafar

**Affiliations:** a Department of Biomedical Engineering and Sciences, School of Mechanical and Manufacturing Engineering, National University of Sciences and Technology, Islamabad, Pakistan; b Department of Research and Development, Islamabad Diagnostic Center (IDC), Islamabad, Pakistan; c Clinical Department of Neurology, University Medical Centre Göttingen and the German Centre for Neurodegenerative Diseases (DZNE), Göttingen, Germany

**Keywords:** Alzheimer’s disease, Creutzfeldt-Jakob disease, incidence, rapidly progressive Alzheimer’s disease, rapidly progressive dementia

## Abstract

Rapidly progressive dementias (RPDs) are a type of fatal dementias that cause rapid progression of neuronal dysfunction. This study aimed to assess the prevalence of APOE genotypes (ε2, ε3, ε4) and PRNP mutations (E200K, M129V) in the general population of Pakistan because of their association with RPDs, including Rapidly Progressive Alzheimer’s Disease (rpAD) and Creutzfeldt-Jakob Disease (CJD). Blood samples (*n* = 100) were collected from healthy Pakistani population and the stated mutations were assessed using polymerase chain reaction. In the analysis of the APOE genotype, ε3/ε3 genotype was the most common (95%), followed by ε3/ε4 (5%) and ε2 allele was completely absent. A low frequency of ε4 allele and the absence of a protective ε2 allele is associated with an increased risk of rpAD. In the case of PRNP mutations, the most common genotype was M129-Ε200 (71%) and V129-Ε200 (29%). E200K mutation was completely absent from the given population. It is noteworthy that the MM homozygous genotype was present in 71 samples, VV genotype was present in 29. Homozygosity on codon 129, as observed in most of our samples, has been associated with more efficient production of PrP^Sc^ and disease pathology. This study provides preliminary data indicating that rpAD and CJD pose a significant threat to the Pakistani population.

## 1. Introduction

Rapidly progressive dementias (RPDs) are categorized as cognitive dysfunction that occurs over the course of months and sometimes even within days [1]. There are various subtypes of RPDs, but in this study, we have only focused on rapidly progressive Alzheimer’s disease (rpAD) and Creutzfeldt-Jakob Disease (CJD). rpAD is characterized by rapidly progressive cognitive dysfunction with dementia that develops within 1–2 years of the disease onset. Rapid progression has roughly been defined as a decline of 6 mini-Mental State Examination (MMSE) points per year and a disease duration of less than two years [2]. Furthermore, amid the increasing range of genetic risk factors that have been identified, the Apolipoprotein E (APOE) gene is highlighted as the strongest and most prevalent, influencing over fifty percent of all instances of Alzheimer’s disease (AD) [3]. The APOE gene is found to be polymorphic at two single nucleotides which includes rs429358 and rs7412. The polymorphism generates three alleles ε2, ε3, and ε4 and six APOE genotypes [4]. The probability of the development of AD in people homozygous for ε4 allele is greater than the ones heterozygous for the allele [5]. The ε2 allele has a protective role against AD so individuals carrying ε2 allele have decreased risk of developing AD [6]. In a study carried out to assess the rate of occurrence of ε4 allele in people with rpAD, the results showed that only 38% of the patients had ε4 allele in contrast to AD patients and none of them was found to be homozygous for ε4 allele, this indicates that RpAD is associated with low frequency of ε4 allele [7].

Additionally, CJD is a rare and progressive neurodegenerative disorder that is life-threatening [8]. The worldwide incidence of CJD is 1 case per million [9]. CJD has three subtypes sporadic CJD, iatrogenic CJD and genetic CJD (gCJD). Sporadic CJD is the most prevalent subtype with 85% of all CJD cases while Iatrogenic CJD accounts for only 1–2% of all cases. gCJD is caused by mutations in the PRNP gene on chromosome 20 and constitutes 10–15% of all CJD cases [10]. More than 55 mutations of gCJD have been identified globally. One of the most common mutations in gCJD patients is E200K (substitution of glutamine to lysine), which has been reported to cause clustered cases globally [11]. Clinical representation of gCJD depends upon the polymorphism at codon 129. The presence of methionine at codon 129 shows greater susceptibility to CJD as compared to valine [12]. CJD’s long incubation period (10–12 years, and in some cases as long as 40 years) and short disease duration (3–12 months) make it difficult to diagnose, which has contributed to a higher death toll since its occurrence [13,14].

The incidence of dementia is increasing in Pakistan, but little to no data is available to remedy the situation. Even though the presence and absence of the APOE4 gene are strongly associated with the development of AD and rpAD respectively, no study has been carried out to check the prevalence of APOE genotype frequency in Pakistani population with respect to RPDs. Similarly, no study has been carried out in the Pakistan to identify the various PRNP mutations associated with CJD. The general population also seems to be unaware of both diseases, and people are hesitant to report the disease because they believe that dementia is a normal part of ageing. Therefore, the aim of this study is to assess the frequency of APOE genotypes and PRNP mutations in the population of Pakistan.

## 2. Methodology

### 2.1. Genotyping

For genetic screening of APOE genotype and PRNP mutations blood samples (*n* = 100) were collected from a healthy population in collaboration with Islamabad Diagnostic Center with the approval of the local ethical review committee (IDCERB10202309). The study was designed in accordance with the Declaration of Helsinki. The age range of the participants was between 15–64 years. Samples were collected at random from both genders without any bias. DNA was extracted from the samples using a commercially available DNA extraction kit (Solar Bio Catalogue number: D1800, China). Primers (Table 1) for amplification of APOE and PRNP alleles were obtained through a comprehensive literature review and were validated through BLAST, primers for PRNP alleles were used in combination i.e., M129-E200 (primer 1 and 3), M129-K200 (primer 1 and 4), V129-E200 (primer 2 and 3) and V129-K200 (primer 2 and 4) [15,16]. The APOE genotype and PRNP mutations were determined by polymerase chain reaction (PCR). A total of 12.5 μl of PCR master mix (Wizbio Solutions, cat#W1401–2, South Korea), 8.5 μl of Nuclease free water, 1 μl of forward primer, 1 μl of reverse primer and 2 μl of DNA template were added in the PCR tube to make 25 μl of total volume. The PCR cycling conditions comprised an initial denaturation phase at 94°C for 3 min, succeeded by 35 cycles, each featuring a denaturation step at 94°C for 30 secs, an annealing phase at varying temperatures (specified in Table 1) for 35 secs, an elongation stage at 72°C for 45 seconds, and a final extension period at 72°C for 7 min. Analysis and visualization of PCR product were done through gel electrophoresis and ChemiDocTM XRS (Bio-Rad, serial number:721BR19365) respectively.

**Table 1.** Table shows the primers used for the amplification of APOE and PRNP genotypes.

Name	Primer Sequence	Temperature (°C)
**Primers for APOE Genotypes**		
ε2 Forward	GCGGACATGGAGGACGTGT	56°C
ε2 Reverse	CCTGGTACACTGCCAGGCA	
ε3 Forward	CGGACATGGAGGACGTGT	57°C
ε3 Reverse	CTGGTACACTGCCAGGCG	
ε4 Forward	CGGACATGGAGGACGTGC	59°C
ε4 Reverse	CTGGTACACTGCCAGGCG	
Name	Primer Sequence	Temperature (°C)
**Primers for PRNP Genotypes**		
M129 Forward (Primer 1)	GGCCTTGGCGGCTACA	57.8
V129 Forward (Primer 2)	GCCTTGGCGGCTACG	55.8
Ε200 Reverse (Primer 3)	CCATCATCTTAACGTCGGTCTC	57.6
K200 Reverse (Primer 4)	CCATCATCTTAACGTCGGTCTT	56.9

### 2.2. Sanger sequencing

In order to validate the results of genotyping, a few initial samples were sequenced via the automated Sanger sequencing method [17]. A total of 30 μl PCR product containing 50 ng genomic DNA was taken and purified to remove contaminants and other impurities using Qiagen PCR cleanup kit, chain termination PCR was performed on 5 μl DNA. The PCR cycling conditions included initial denaturation at 98°C for 4 min, followed by 35 cycles at 98°C for 10 secs, after that annealing was done at 60°C for 30 secs, extension at 72°C for 40 sec and final extension at 72°C for 10 min. Each band in the capillary gel was read by the computer, and fluorescent tags in each band were excited by laser resulting in the emission of light which was detected by the computer. The output was seen on the chromatogram, on which different coloured waves represented different bases.

## 3. Results

### 3.1. Genotyping

**Figure 1.**
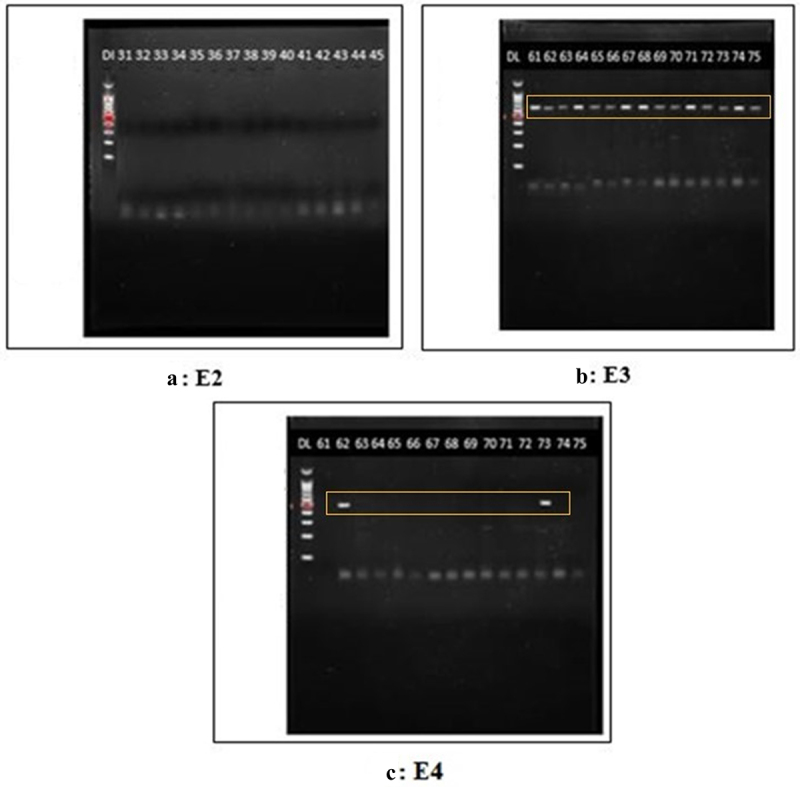
Representative gels for ε2, ε3, and ε4. Gel a shows that no band could be seen for ε2 allele, therefore ε2 allele was absent in all 100 samples. Gel B shows that ε3 allele was present in all the 100 samples in homozygous pattern, except for 5 samples in which it is present in heterozygous pattern with ε4 allele. Gel C shows that ε4 was present in only 5 samples in heterozygous pattern ε3/ε4.

**Figure 2.**
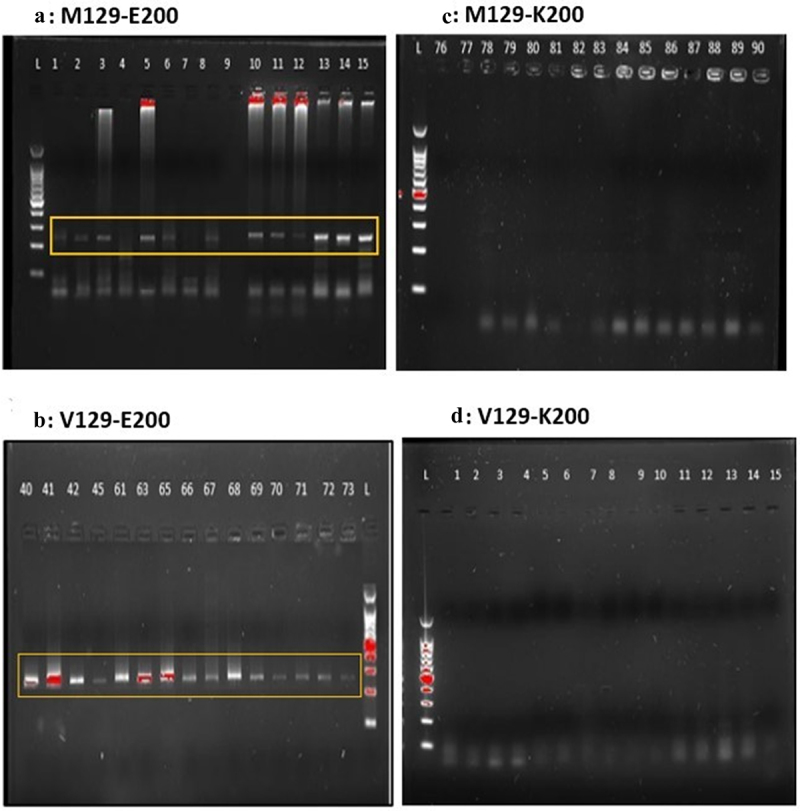
Representative gels for healthy and mutated sequence. Gel A and B shows the presence of M129-Ε200 and V129-Ε200 in the sample (*n* = 100) whereas gel C and gel D shows the absence of mutations i.e., M129-K200 and V129-K200.

As depicted in Table 2 and 3, the most prevalent allele in 100 samples was ε3, it was present in all the samples with an allelic frequency of 0.975 (97.5%). The least prevalent allele was ε2, which was completely absent from the sample with an allele frequency of 0. ε4 allele was present in 5 samples with an allele frequency of 0.025 (2.5%). The most prevalent genotype was ε3/ε3 with a genotype frequency of 0.95 (95%). ε2/ε2, ε2/ε3, ε2/ε4, and ε4/ε4 genotypes were completely absent. The ε3/ε4 genotype was the second most prevalent with an allele frequency of 0.05 (5%). In the case of PRNP mutations, the genotype M129-Ε200 emerged as the most commonly observed genotype, exhibiting a frequency of 0.71 (71%). Following closely behind, the genotype V129-Ε200 manifested as the second most prevalent combination, with a frequency of 0.29 (29%). The genotypes V129-K200 and M129-K200 were not detected in any of the samples. The representative gels for the APOE genotype and PRNP genotype are shown in Figures 1 and 2 respectively.

**Table 2.** Allele frequency.

Sr No.	Alleles	Number of alleles present in n	Ratio of no. of allele present in n to total no. of alleles (200).	Allele frequency
**Frequency of APOE and PRNP alleles *n*=100**				
1.	ε2 (protective)	0	0/200	0
2.	ε3 (neutral)	195	195/200	0.975
3.	ε4 (AD, RpAD)	5	5/200	0.025
4.	M129-Ε200 (healthy)	171	171/200	0.855
5.	V129-Ε200 (healthy)	29	29/200	0.145
6.	M129-K200(mutant)	0	0/200	0
7.	V129-K200 (mutant)	0	0/200	0

The table shows the allele frequency of APOE and PRNP alleles in the selected dataset (*n* = 100). ε2 allele was completely absent from the population. The ε3 allele in ε3/ε3 genotypic combination was the most prevalent. The ε4 allele was only present in ε3/ε4 combination. Results also indicated the absence of mutation at codon 200. The most prevalent genotype was M129-Ε200 (71%) and V129-Ε200 (29%) whereas M129-K200 and V129-K200 were absent in the subjects.

**Table 3.** Genotypic distribution.

Sr No.	Genotype	Number of individuals	Ratio of genotype to total	Genotype Frequency
**Homozygosity and Heterozygosity of APOE and PRNP Genes**				
1.	ε2/ε2	0	0/100	0
2.	ε2/ε3	0	0/100	0
3.	ε2/ε4	0	0/100	0
4.	ε3/ε3	95	95/100	0.95
5.	ε3/ε4	5	5/100	0.05
6.	ε4/ε4	0	0/100	0
7.	M/M	71	71/100	0.71
8.	M/V	0	0/100	0
9.	V/V	29	29/100	0.29
10.	E/E	100	100/100	1
11.	E/K	0	0/100	0
12.	K/K	0	0/100	0

The table shows the genotypic distribution, ratios, and frequencies of APOE polymorphism and PRNP gene in a study population of 100 individuals. For the APOE gene, ε3/ε3 was the predominant genotype (95%), with ε3/ε4 observed in 5%; other genotypes were absent. For the PRNP gene, codon 129 revealed a majority of individuals with the M/M genotype (71%), followed by V/V (29%), with no heterozygous M/V genotypes.

### 3.2. Sanger sequencing

The PCR products were sequenced. The sequencing results were confirmed using BLAST (https://blast.ncbi.nlm.nih.gov/Blast.cgi). The sequences acquired using Sanger sequencing are shown in Figure S.1 of supplementary data.

## 4. Discussion

The presence of different isoforms of the APOE gene in different genotype combinations determines the susceptibility of the person to developing AD in the future. The ε2 gene has a protective role against AD, with a worldwide prevalence of 8.4% in healthy individuals and 3.9% in AD patients [6]. The ε2 allele is known to reduce Aβ pathology in humans, the autopsy of AD patients carrying the ε2 allele showed a lower density of Aβ-containing senile plaques when compared to ε3ε3 [18]. PET imaging also confirmed that Aβ accumulation happens at a much lower rate in non-demented individuals carrying ε2 allele as compared to ε3ε3 homozygotes [19]. In this study, the allele and genotype frequencies of the ε2 allele were found to be 0. Therefore, the absence of ε2 allele in non-demented individuals indicates that they are deprived of the protective role that may have been provided by ε2 if present. This increases the risk of developing AD in the population. ε3 is the most common isoform of the APOE gene, with a prevalence of 77.9% worldwide [20]. It is believed to play a neutral role with respect to AD [21]. In this study, the findings indicate that all 100 samples contain the ε3 allele. The allele frequency of the ε3 allele in 100 samples was 100%. The most common genotype in the subjects was ε3ε3 with 95% prevalence, the second most common genotype was ε3ε4 with 5% prevalence. Although ε3 does not play any role in the development of AD, the absence of protective ε2 and the presence of ε3 with ε4 indicates that there is a relatively high risk of AD among the subjects as compared to if they had the ε2 allele.

The ε4 allele is the major risk factor for AD and the second most prevalent isoform of the APOE genotype after ε3. The allele frequency of the ε4 allele among the general population worldwide is 13.7% and among AD patients, the allele frequency is 36.7% [22]. The probability of AD in individuals with one ε4 allele increases by 2–3-fold while individuals who have two ε4 alleles in the homozygous pattern. have a 10–15-fold increased risk of developing AD. ε4 allele is involved in exacerbation of Aβ deposition [23]. The current study indicated that the most common genotype in the subjects was ε3ε3, with 95% prevalence. The ε4 is the major risk factor for AD, with a global prevalence of 13.7%, and is involved in exacerbating Aβ deposition [22]. In our dataset, the ε4 allele was present in the ε3/ε4 pattern only with a 5% genotype frequency, which suggests that 5% of the subject population is at risk of developing AD. While a high frequency of the ε4 allele increases the risk of AD, a decreased frequency is believed to increase the risk of rpAD [24]; however, a full consensus on this theory has not yet been reached. Therefore, if the allele frequency of the ε4 allele is lower, the chance of developing rpAD increases. With the complete absence of the protective ε2 allele and the presence of the ε4 allele in heterozygous conditions, the chance of developing rpAD increases.

PRNP mutations are associated with the onset of genetic gCJD. The global incidence of gCJD is estimated to be 10–15%. Among these mutations, the E200K mutation, characterized by a substitution from lysine to glutamic acid, stands out as the most common and extensively researched mutation, occurring annually at a rate of 0.27 per million [25].

The study indicated that the mutation from E200 to K200 was not found in any of the participants, which confirms the rarity of this mutation. On the other hand, the presence of homozygous methionine at codon 129 is a risk factor that facilitates the formation of PrP^c^/PrP^Sc^ complexes, causing rapid neurodegeneration if present in homozygous conditions [26]. The occurrence of methionine at codon 129 is found to be the highest globally from 60%-80% in the European population to 60%-70% in the Asian population. Likewise, the prevalence of E200 is 90–95% in the European population and Asian populations. Similarly, our study revealed that the most prevalent allelic form was M129-E200, which corresponds to homozygous methionine at codon 129 (0.71%) signifying that individuals with this combination may have higher susceptibility towards CJD due to the presence of methionine (MM) at codon 129, whereas presence of E200 allele does not modify risk substantially, its presence does not mitigate the increased risk associated with 129 allele [27,28]. The frequency of V129 (MV or VV) allele is rare. In Asian populations V129 is below 1% and in European populations is approximately 1–3% highlighting its essential role in understanding gCJD and other prion diseases. The presence of valine at codon 129 signifies a lower to intermediate risk of CJD as it gives rise to a more stable and less pathogenic form of prion protein, hindering the process of PrP^c^/PrP^Sc^ complex formation. Similarly, K200 is relatively rare, with 2–5% global occurrence. The presence of K200 is associated with an increased risk of gCJD when combined with the primary risk factor Methionine at codon 129 [29]. Our study aligns with the study as V129- K200 and M129-K200 are absent from the population indicating the rarity of this mutation. Conversely, the second most predominant genotypic expression found in our study V129-E200 i.e., 0.29, indicates a lower risk of developing CJD as compared to a combination involving M129.

This study has some limitations, the genotyping analysis has a relatively small sample size, which restricts the generalizability of our findings. One of the limitations of our study is the inability to perform DNA sequencing on all samples due to its high cost. Future research could benefit from larger sample sizes in order to improve genetic association detection, increase the reliability of results, and conduct a more thorough subgroup analysis.

## 5. Conclusion

Our findings identified the absence of the protective ε2 allele, the presence of the neutral ε3 allele in the majority of the population and the ε4 allele in five samples indicating the risk of AD and rpAD. The presence of M129-E200 indicates a higher susceptibility to CJD due to the presence of methionine. E200 does not significantly change the risk. V129-E200 is consistent with its rare occurrence globally. Even though the study targeted a smaller sample size with a less diverse group, it can act as an essential preliminary step for conducting research studies for a larger sample size with a more diverse population. Therefore, it is essential to identify and report cases to devise correct diagnoses and treatments to curtail this disease.

## Supplementary Material

Supplemental Material

## Data Availability

Data supporting the findings of this study are available from the corresponding author upon reasonable request.
