# Supplementary material for: Genetic assessment of apolipoprotein E polymorphism and PRNP genotypes in rapidly progressive dementias in Pakistan
Source: Prion. 2024 Dec 9;18(1):103–9. doi: 10.1080/19336896.2024.2439598 (PMC11812391; doi:10.1080/19336896.2024.2439598)
Supplement: Supplemental Material [file KPRN_A_2439598_SM9687.docx]

**Supplementary Figure S.1:**


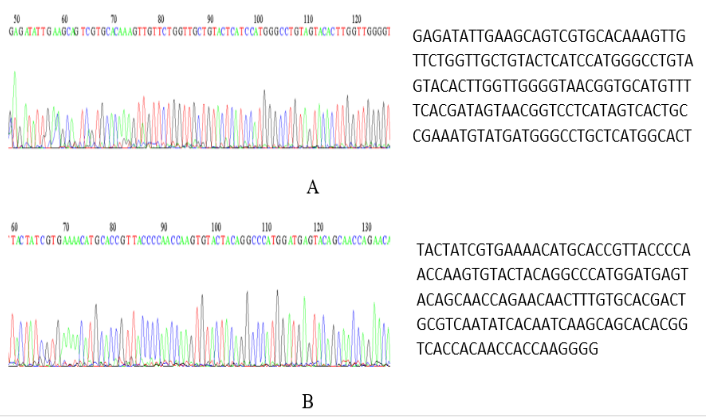


ALT text for supplementary figures

1. Figure S.1: Chromatogram of E200 and M129 genes via Sanger sequencing. A) E200 amplification. B) M129 amplification.
